# Support from the Internet for Individuals with Mental Disorders: Advantages and Disadvantages of e-Mental Health Service Delivery

**DOI:** 10.3389/fpubh.2014.00065

**Published:** 2014-06-11

**Authors:** Jörn Moock

**Affiliations:** ^1^Innovation Incubator, Leuphana University Lüneburg, Lüneburg, Germany

**Keywords:** public mental health, mental disorders, e-mental health, mobile support, computerized cognitive behavioral therapy

## Abstract

Mental disorders are common in almost all industrialized countries and many emerging economies. While several trials have shown that effective treatments exist for mental disorders, such as pharmacotherapy, psychological interventions, and self-help programs, the treatment gap in mental health care remains pervasive. Unrestricted access to adequate medical care for people with mental disorders will be one of the pressing public mental health tasks in the near future. In addition, scarcity of financial resources across the public mental health sector is a powerful argument for investigating innovative alternatives of delivering mental health care. Thus, one challenge that arises in modern mental health care is the development of innovative treatment concepts. One possibility for improving mental health care services is to deliver them via the Internet. Online-based mental health services have the potential to address the unmet need for mental health care.

## Introduction

Mental disorders are common in almost all industrialized countries and many emerging economies. Wittchen and colleagues (1) reported that among European Union (EU) countries, 38.2% of the population suffered from a mental disorder each year. Among Americans aged 18 years and older, the figure was reportedly 26.2% annually (2). Mental disorders are responsible for high rates of work incapacity (3) and therefore generate huge costs to society (4, 5). Those suffering from such disorders report a reduced quality of life and an enormous burden of disease. Furthermore, their lives are often plagued with discrimination and social stigma (6, 7) as well as a greater risk of educational difficulties, social problems, vulnerabilities to abuse, and additional health problems (8).

Causes for this steady increase could lie in the change from manual labor in the twentieth century toward knowledge-based work in the twenty-first century (9). There has been an extensive discussion regarding the rise of mental disorders in the last few decades. Rössler pointed out the impact of developments within the diagnostic system of mental disorders (10), while other researchers indicated cultural reasons, such as a more pessimistic attitude toward life than found in earlier generations (11). Other scientists discussed over-diagnosis of psychiatric disorders and the resultant increase in psychotropic drug prescriptions as the cause (12).

While several trials have shown that treatments such as pharmacotherapy, psychological interventions, and self-help programs are effective, the treatment gap remains pervasive in most industrialized countries. The proportion of individuals who actually receive treatment in the specialized mental health care system or in the general health care system is low (13), and initial intervention after onset is frequently delayed for many years (14). In the US, approximately 67% of the persons suffering from mental disorders are not treated (15). In the EU, the figure is 74.0%, yet the treatment gap in diabetes is only 8.0% (16). Classical individual reasons for failing to seek help, and thus facing an unmet need are: the burden is not perceived, a belief that the problem is only a temporary phenomenon, and a decision to deal with the burden of disease without outside assistance (17). Social barriers to receiving effective treatment comprise a critical shortage of skilled psychotherapists as well as psychiatrists, resulting in long waiting lists and increasing costs (18, 19).

Unrestricted access to adequate medical care for people with mental disorders will be one of the pressing public mental health tasks in the near future. In addition, scarcity of financial resources across the public mental health sector is a powerful argument for investigating innovative alternatives of delivering services. Thus, one challenge that arises is the development of innovative treatment concepts.

One possibility for improving services is to deliver them via the Internet. Online-based mental health services have the potential to address the treatment gap. Health care providers and health insurance companies realize that a cost-effective solution is to use the Internet to provide services. In fact, it could be the initial point of service, fostering a cultural change in mental health services and mental health literacy, empowering clients with greater control by managing self-diagnosed conditions. Whether this can be achieved is unknown. The Internet is an area of continuous development and innovation that should be used for distribution of innovative and effective treatment programs and self-management tools in the field of public mental health.

Against this background, we discuss mental health service delivery via the Internet in more detail as one approach to complement existing “real life” mental health care services. We also highlight best practice examples of how different participants join in the sector and the endless opportunities of the Internet.

## The Internet as a Public Mental Health Intervention and Prevention Tool

The Internet has changed nearly every aspect of our lives over the past decades. In early years, only a few purely text-based websites existed, but currently, more than 14 billion web pages are available. This rapid development has paved the way for a communication platform on which digital media content can be delivered to a wide variety of mobile devices, such as desktop computers, wireless laptops, and smartphones.

There is a proliferation of mobile devices that offer innovative opportunities to support persons suffering from mental disorders. The shift from old mediums of information dissemination to mobile technology has been lightning fast and the global penetration of smartphones and tablets is amazing. Yet, the number of adults integrating the Internet into their daily lives is still growing.

**Figure 1:**
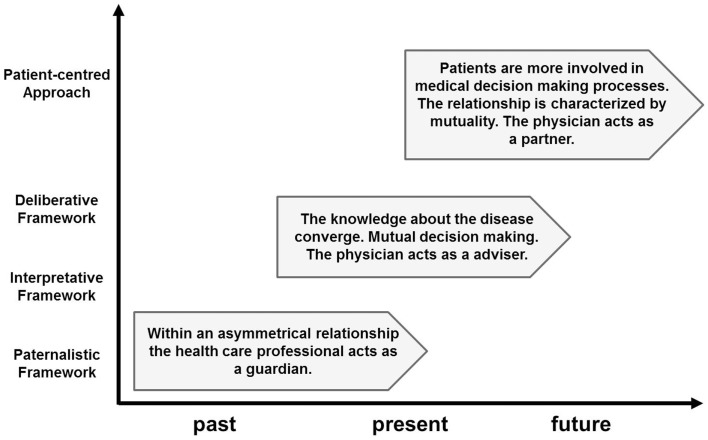
**Overview of conceptual frameworks for patient-professional communication**.

The Internet has become a basic tool for trading, entertainment, communication, education, and knowledge transfer. And, with the increasing availability of fast broadband access, there are multifaceted attributes that can foster mental illness interventions and prevention programs (20). These attributes include anonymous, easy, and flexible access to health care providers 24 h a day, and in increasingly remote areas. Additionally, from an economic perspective, it could be an important cost-effective delivery mode because it uses fewer personnel and less infrastructure (21, 22). The opportunity for bidirectional communication allows a more active role for service consumers in shaping the support available to them (Figure 1).

On the technical side, a big challenge of providing customized programs is the deployment of all the capabilities of the Internet. Recently, the Institute for Healthcare Informatics counted more than 40,000 mobile apps related to health care, available for download from the US iTunes App Store (23). In the near future, patients’ use of such apps in addition to or superseding conventional treatment programs will increase. However, mobile health care apps will first have to clear some hurdles, including patients’ refusal because of breach of data protection and physicians’ fear of getting overloaded with vital-sign data generated by remote monitoring and tracking apps.

## What is e-Mental Health Care?

In a technical context, e-mental health care, a relatively new term, is a practice supported by electronic processes and communication. It can include mental health applications and links on mobile phones. e-Mental health services can include information, peer support services, computer- and Internet-based programs, virtual applications, and games, as well as real time interaction with trained clinicians. A more content-oriented definition is provided by the National Health Service (NHS) Network of the NHS Confederation (24): “e-Mental health is the use of information and communication technologies to support and improve mental health, including the use of online resources, social media, and smartphone applications.” The Trimbos Institute (Dutch Institute of Mental Health and Addiction) defines e-mental health as an information and communication technology to support or improve mental health and health care. e-Mental health has the potential to provide advantages such as low cost, easy access, and anonymity for users (25, 26). However, there are also a number of unsolved challenges regarding user privacy and confidentiality.

## e-Mental Health: Opportunities for Mental Health Care Services

The mental health care sector is developing web-based solutions for persons suffering from mental disorders by using different methods and strategies. Various e-mental health services and their benefits are presented in the following sections.

### Informed mental health care clients

Nowadays, people use the Internet to get information about symptoms or treatment programs and health care services, and they compare treatment options. For example, the NHS website “NHS Choices”^1^ is one of the most popular health websites in the UK. The website mentalhealthminute.com, funded by a grant from the Canadian Council on Learning and sponsored in part by the Canadian Alliance on Mental Illness and Mental Health, is responsible for providing information about mental health in everyday language.

### Peer support

We all experience peer support everyday, from telling our best friend our worries and fears to discussing the weather forecast with our neighbor. The Internet can connect people separated by time and space who might not meet otherwise, and the benefits include opportunities for social support and access to sources of information. The removal of social cues and social distinctions such as disability, race, and facial expressions through text-only communication can help the shyest people feel more confident about communicating with others. People can learn through sharing or questioning information, verbalizing opinions, weighing arguments, and active learning (27). Independent portals such as “Patient Opinion”^2^ in the UK provide a platform for patients to publish their stories about health care services. In Germany, “Diskussionsforum Depression”^3^ offers a portal for one specific disorder in the field of mental disorders. The primary function of this forum is to provide a space where mutual support is given and received by anyone affected by depression. Other unmoderated online sources such as Twitter or Facebook are also frequently used to exchange information or to seek help.

### Treatment programs for mental disorders

There are other e-mental health programs available for several mental disorders. Furthermore, these treatment programs have been evaluated in randomized controlled trials (21, 28–36). Available e-mental health programs and applications range from self-help services (28) to treatment programs based on structured computer-administered therapy with or without computer-generated feedback, and web-based interventions with therapist support (29, 30). In addition, a few programs use video conferencing tools as a communication platform between patient and therapist (37).

e-Mental self-help services enable users to learn more about their mental health condition and empower them to strengthen their self-management and improve their health, sometimes including peer-to-peer support. Self-help is useful at any stage of recovery and assists professionals with improving the care they provide. The majority of e-mental health treatment programs provide cognitive behavioral therapy (CBT) skills (38), and there is increasing evidence that self-help interventions are effective for reducing symptoms of anxiety disorders (39). Recent studies have shown that therapist-guided web-based treatment programs can be effective compared to conventional care (28, 34, 40, 41), and a randomized controlled trial in Australia suggested that unguided web-based interventions can be effective in reducing depressive symptoms (42). However, in a meta-analysis, Spek and colleagues (43) analyzed 12 such trials examining the effectiveness of computerized cognitive behavioral therapy (c-CBT) interventions, and found that they showed little evidence that un-guided Internet-based self-help services improved mental well-being. Furthermore, little is known about the cost-effectiveness, practicability, and acceptability, and further research is required to evaluate the economic and social impact of these interventions.

In addition to content-specific criteria, interest in the economic exploitability of c-CBT programs has increased. As Griffiths and colleagues report, several c-CBT intervention programs are commercial and neither freely available nor part of common mental health care services. Among English programs, the e-hub group at the Australian National University provides information and access to self-help services free of charge^4^. Its objective is to provide information and access to self-help services based on reviews of scientific literature (44). Currently, it comprises four programs. Briefly, “BluePages”^5^ gives evidence-based information on symptoms, diagnosis, treatment, and the experience of depression. “The MoodGYM”^6^ is an un-guided self-help CBT program for depression comprising 5 modules and 29 exercises. The e-couch tool^7^ offers evidence-based information and automated self-help skills training for depression, generalized anxiety disorder, and social anxiety disorder. Finally, the “BlueBoard”^8^ is a moderated peer-to-peer support portal for people experiencing or caring for people with depressive, bipolar, or anxiety disorders.

Recently, the National Institute for Health and Care Excellence (NICE) has updated their review on c-CBT programs for depression and anxiety. They approved “Beating the Blues”^9^ for treating depression, anxiety, and phobias, and “Fear Fighter for Panic and Anxiety”^10^ for use with individuals who suffer from phobias or panic attacks.

In the Netherlands, United Kingdom, Scandinavian countries, Australia, and New Zealand, guided and un-guided self-help mental health services are currently being integrated into the health care system. In other countries such as Germany and France, discussions about implementing e-mental health resources into the health care system are taking place.

## Disadvantages and Concerns

The availability of mobile health apps for use on smartphones continues to increase. However, it is an ongoing debate whether the use of such technologies is a boon or bane, and evidence about efficacy or effectiveness is scarce for most apps. Thus, there is a need to address concerns and start a debate on the pros and cons of e-mental health services delivered this way.

First, heavily-used social networking sites such as MySpace and Facebook are linked to a decline in mental well-being (45). However, social interaction is an essential human need (46) and social networks provide an invaluable resource of possible social connections. At first glance, it is counterintuitive that interacting with web-based social networks would deteriorate mental well-being, but social interaction is more than words; it also includes confidence, emotions, physicalness, and many other aspects of human living. These could foster a gain in diagnosable mental disorders and online-based programs could be an add-on, not a substitute, for “real life” mental health care services. Second, there is a need for valid and reliable methods of evaluating digital treatments and therapies. A promising start for effective and valid research methods could be mixed-method approaches and qualitative research. Third, there is a regulatory challenge to provide mobile app designers and developers clear and well-documented guidance to help them develop tools that coincide with the needs of regulatory authorities such as the Food and Drug Administration, the Federal Joint Committee, NICE, Therapeutic Goods Administration, and Medsafe. The Food and Drug Administration has published recommendations providing health app developers an authorized roadmap for developing mobile health care apps as medical devices appropriate for self-management or treatment (47). Fourth, evidence-based recommendations are needed to prevent those who seek medical information and support from being misled by uncertified self-management tools and treatment programs.

While online therapy offers access to mental health information and treatment options, several disadvantages must be discussed. For example, while online mental health services eliminate geographic restraints, enforcement of legal and ethical codes becomes difficult, since many states have different licensing requirements and treatment guidelines. Also, most statutory insurance policies do not cover online therapy, making it ineligible for reimbursement and thus, an out-of-pocket service for the majority of the population. And, because most online mental health care services are not regulated by governance, confidentiality and privacy must be questioned. In addition, unlike “real life” therapy, therapists likely cannot respond adequately to crisis situations. Finally, online therapy is suitable primarily for persons with mild or moderate mental disorders and is inappropriate for those with serious psychiatric disorders such as schizophrenia.

## Discussion

Mental disorders are important, but until recently have received limited attention in the global health discussion. Public mental health, as a young scientific discipline grounded between medical practice, life sciences, and humanities, is an attempt to improve on this. One traditional goal of public (mental) health is the promotion of population (mental) health (48). Public e-mental health offers the opportunity to raise awareness of healthy behaviors and mental health care programs on a population level. From a public mental health perspective, it could offer amenities to all persons suffering from mental disorders.

e-Mental health treatment programs and services are available for a wide range of mental disorders. Several treatment programs have been rigorously evaluated and some are recommended by independent scientific institutes such as NICE. There is evidence that e-mental health services are effective as intervention tools, and further, as prevention tools (43, 49). These programs can reach patients, regardless of their time or location, who could not previously be reached by conventional means. A further benefit for the public sector is the ability to reach people who cannot currently be fully realized by conventional methods. Patients often experience stigma, discrimination, and the associated social isolation (50), and these marginalized groups have a close interest in using the Internet as a platform for mental health care services.

Findings of randomized controlled trials conducted in Europe, Australia, New Zealand, and the US (21, 28, 35, 36, 43, 51, 52) have demonstrated that web-based public mental health care is an option in treating a wide range of mental disorders, showing that online mental health programs have the potential to reach those with an unmet need for health care services.

### e-Mental health implementation process

Health care consumers, providers, decision makers, and insurance companies have a large interest in change. e-Mental health should be an integral part of a mental health strategy in the future. Most e-mental health programs focus merely on capturing information about health status, and activities are linked directly to the activities of the client, but it can offer bidirectional communication, and health care providers can influence mental health behavior changes and monitor mental disorder self-management in a context-sensitive way. Real time e-mental health can make it possible to collect and analyze such context-specific information to help individuals directly, without a time lag. Much of the ongoing research attempts to tackle all the opportunities and concerns, but more research is needed in this area.

## Conclusion

Online assisted health services for individuals with mental disorders will not resolve all the challenges associated with the lack of availability or long waiting lists. However, they can pull their weight as alternatives to conventional treatment programs and enable available resources to get more mental health patients into therapy in spite of the fact that several issues are raised. It appears that, in spite of much uncertainty about the impact of e-mental health on the efficiency and effectiveness of mental health services, health care providers are able to supply more clients using fewer resources through the use of e-mental health. It remains to be seen whether the dissemination of e-mental health care resources will meet all the expectations.

## Conflict of Interest Statement

The author declares that the research was conducted in the absence of any commercial or financial relationships that could be construed as a potential conflict of interest.

## Footnotes

^1^http://www.nhs.uk

^2^http://www.patientopinion.org.uk

^3^http://www.diskussionsforum-depression.de/diskussionsforum/

^4^http://www.ehub.anu.edu.au/welcome.php

^5^http://www.bluepages.anu.edu.au

^6^http://www.moodgym.anu.edu.au

^7^http://www.ecouch.anu.edu.au

^8^http://www.blueboard.anu.edu.au

^9^http://www.beatingtheblues.co.uk

^10^http://www.fearfighter.com
